# Sampling fish gut microbiota ‐ A genome‐resolved metagenomic approach

**DOI:** 10.1002/ece3.70302

**Published:** 2024-09-17

**Authors:** Eiríkur A. Thormar, Søren B. Hansen, Louise von Gersdorff Jørgensen, Morten T. Limborg

**Affiliations:** ^1^ Globe Institute, Faculty of Health and Medical Sciences, Center for Evolutionary Hologenomics University of Copenhagen Copenhagen K Denmark; ^2^ Section for Parasitology and Aquatic Pathobiology, Department of Veterinary and Animal Sciences, Faculty of Health and Medical Sciences University of Copenhagen Frederiksberg C Denmark

**Keywords:** fish metagenomes, genome‐resolved metagenomics, sampling fish microbiomes, shotgun metagenomic sequencing, zebrafish gut microbiota

## Abstract

Despite a surge in microbiota‐focused studies in teleosts, few have reported functional data on whole metagenomes as it has proven difficult to extract high biomass microbial DNA from fish intestinal samples. The zebrafish is a promising model organism in functional microbiota research, yet studies on the functional landscape of the zebrafish gut microbiota through shotgun based metagenomics remain scarce. Thus, a consensus on an appropriate sampling method accurately representing the zebrafish gut microbiota, or any fish species is lacking. Addressing this, we systematically tested four methods of sampling the zebrafish gut microbiota: collection of faeces from the tank, the whole gut, intestinal content, and the application of ventral pressure to facilitate extrusion of gut material. Additionally, we included water samples as an environmental control to address the potential influence of the environmental microbiota on each sample type. To compare these sampling methods, we employed a combination of genome‐resolved metagenomics and 16S metabarcoding techniques. We observed differences among sample types on all levels including sampling, bioinformatic processing, metagenome co‐assemblies, generation of metagenome‐assembled genomes (MAGs), functional potential, MAG coverage, and population level microdiversity. Comparison to the environmental control highlighted the potential impact of the environmental contamination on data interpretation. While all sample types tested are informative about the zebrafish gut microbiota, the results show that optimal sample type for studying fish microbiomes depends on the specific objectives of the study, and here we provide a guide on what factors to consider for designing functional metagenome‐based studies on teleost microbiomes.

## Graphical abstract

Despite a surge in microbiota‐focused studies in teleosts, few have reported functional data on whole metagenomes as it has proven difficult to extract high biomass microbial DNA from fish intestinal samples. Addressing this, we systematically tested four methods of sampling the zebrafish gut microbiota—collection of faeces from the tank, the whole gut, intestinal content, and the application of ventral pressure to facilitate extrusion of gut material—along with water samples as an environmental control to address the potential influence of the environmental microbiota on each sample type. Using genome‐resolved metagenomics and 16S metabarcoding, our analyses indicate that while all sample types tested are informative about the zebrafish gut microbiota, the optimal sample type for studying fish microbiomes depends on the specific objectives of the study, and here we provide a guide on what factors to consider for designing functional metagenome‐based studies on teleost microbiomes.

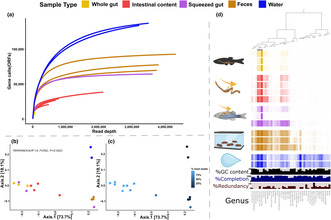

## 1 INTRODUCTION

The application of microbiome data has seen an immense growth in the field of molecular ecology including numerous fish species (François‐Étienne et al., 2023; Minich et al., 2022; Stagaman et al., 2020). However, compared with warm‐blooded animals, there is a remarkable scarcity of microbiome studies on fish that are based on functional insights from whole genome shotgun sequencing techniques. Interestingly, this seems to be partly explained by a significantly lower microbial biomass in fish intestines compared with warm‐blooded animals (Limborg et al., 2023), which has complicated the sampling of microbial DNA as samples often contain >90% host DNA. Here, we argue that part of this challenge can be addressed with an optimised sampling protocol by showcasing the outcome of different protocols to sample a fish gut microbiome community using the zebrafish as a model system.

Zebrafish (*Danio rerio*) is one of the most studied model organisms to date. The use of the model dates back to the research of George Streisinger in the late 1960s and is today used in a wide variety of research topics from evolutionary biology to human health studies (Grunwald & Eisen, 2002). Today's extensive use of the model is well grounded in that zebrafish are relatively easy to rear, cheap to care for, easy to breed, have a generation time of only 3 months, have a well‐defined embryology (Spence et al., 2008), and are readily amenable to genetic modification (Hwang et al., 2013; Tennant et al., 2019). Furthermore, a plethora of zebrafish‐related scientific literature exists with over 53,000 publications to date (PubMed Search Results 2023). It is no surprise then that interest in applying zebrafish as a model organism to study the increasingly popular topic of host–microbiota interactions is on the rise, and a multitude of research papers, preprints, and reviews have been published on the matter, ranging from probiotic effect on behaviour (Davis et al., 2016; Stagaman et al., 2020) to effects of the immune cell development gene, *irf8*, on the zebrafish microbiota (Earley et al., 2018). Expectedly, it seems that the most prevalent method of studying the zebrafish gut microbiota is using 16S metabarcoding, a valuable and powerful tool to investigate and describe the composition, diversity, and taxonomic landscape of the gut microbiota. However, given the increasing interest in the zebrafish model for studying host–microbiota interactions and advances in the development of sequencing technologies and bioinformatic tools, it is interesting to consider the number of studies applying more functional approaches. To date, three publications (Kayani et al., 2021, 2022; Zhang et al., 2021) and a single preprint (Gaulke et al., 2020) appear to form the entirety of the zebrafish short‐read‐based metagenomics literature. Only one of which (Kayani et al., 2022) uses a genome‐resolved metagenomics approach, that is de novo assembly of whole bacterial genomes often termed metagenome‐assembled genomes (MAGs). One of the studies relies on sampling whole intestines (Zhang et al., 2021), while the others all rely on faecal matter sampled directly from the tank to characterise the gut metagenome. However, when faecal matter is collected, an issue with the water microbiota arises. As the sample is acquired directly from the tank, the boundary between the host microbiota and the water microbiota may be blurred, potentially rendering functional and taxonomic insights less than optimal. Although studies on larger fish species using metagenomic shotgun sequencing have included intestinal content and gut mucosal scrapes from various gut regions (Cheaib et al., 2021; Collins et al., 2021; Rasmussen et al., 2023; Riiser et al., 2019), those strategies are less feasible for smaller fish, such as zebrafish, which motivates us to focus on sampling strategies with broader applicability across the teleost species.

This leads to considerations regarding which sampling strategy is best for representing the zebrafish intestinal microbiota for a metagenomic shotgun sequencing study while providing enough microbial DNA reads. There are several different methods noted in the literature for sampling the zebrafish microbiota, (Stagaman et al., 2020) but little tangible consensus on what constitutes an appropriate sampling protocol. We therefore addressed the sampling of the zebrafish intestinal microbiota by comparing four different methods throughout a genome‐resolved metagenomic pipeline. We compared faeces sampled directly from the tank (here “faeces”), whole gastrointestinal tract samples (here “whole gut”), pressure on the underside (here ‘squeezed gut’), and finally intestinal content dissected from whole guts (here ‘intestinal content’). Water samples were included as an environmental control and for estimation of environmental contamination. Differences among sample types were evident on all levels from sampling to analyses. There are more potential sampling techniques that may cover the niches of the zebrafish gut microbiota, that is, differential composition and function in different sections of the gut. We have only addressed the aforementioned sampling techniques with an aim on how to best generate whole metagenome data as they are both easy to apply and representative of the whole zebrafish gut microbiota community.

## 2 METHODS

### 2.1 Zebrafish husbandry and ethics statement

The zebrafish were reared in a re‐circulatory system (Techniplast active blue) with a light/dark cycle of 14/10 h. The zebrafish were fed with dry feed (ZM‐300, ZM‐400, ZM Fish food and Equipment, UK) and live Brine Shrimp (*Artemia* sp., ZM Fish food and Equipment, UK). The zebrafish collected for this study were all housed in the same tank. All gut microbiota samples were obtained from adult zebrafish The experiment was conducted in accordance with a permit from The Animal Experiments Inspectorate under the Danish Ministry of Environment and Food (Permit: 2021‐15‐0201‐00951). For euthanisation of fish, 300 mg/L of tricaine methanesulphonate (MS222, A5040, Sigma‐Aldrich, Denmark) was used.

### 2.2 Sampling

As described above, we aimed to compare four different approaches for their efficiency in sampling DNA for characterising the zebrafish gut metagenome using shotgun sequencing. These sampling methods all differ in degree of invasiveness and relative ease of sampling (Table 1).

**TABLE 1:** Overview of the sampling process among sample types in terms of feasibility of sampling.

	Dissected whole gut 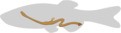	Dissected intestinal content 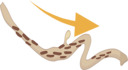	Squeezed gut content 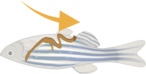	Faeces 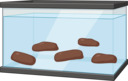
Sample description	Whole gut dissected from fish	Whole gut dissected from fish and intestinal content dissected out	Pressure applied to the abdominal side of the fish from the pectoral fin to the anal fin and the excretion sampled	Faecal matter collected from the bottom of the tank
Invasiveness	High	High	Moderate	None
Ease of sampling	Moderate	Difficult	Moderate	Easy

#### 2.2.1 Whole gut

The whole gastrointestinal tract of zebrafish was carefully dissected out and stored in an MP Biomedicals Lysing E Matrix tube with 0.5 mL of 1X DNA/RNA Shield (Zymo research). A total of three whole gastrointestinal tracts were collected. This is an invasive, and rather time‐consuming technique but does carry the benefit of individual association, consistency, and no direct contact with the surrounding water. The main drawback of this method is that most of the DNA extracted will likely be host DNA, a common feature of fish microbiota studies (Collins et al., 2021; Hennersdorf et al., 2016; Rasmussen et al., 2021; Riiser et al., 2019), and something we have previously observed in whole guts of zebrafish (unpublished data). This is a potential limitation as high host DNA fractions can impact the quality and resolution of the analysis of the microbiota (Pereira‐Marques et al., 2019).

#### 2.2.2 Squeezed gut

We sampled intestinal content from the zebrafish by gently ‘squeezing’/putting pressure along the abdominal side of the fish from the pectoral fin toward the anal fin. We sampled the resulting excretion from the urogenital opening using an inoculation loop, ~ ½ of a 10 μL loop faecal matter was then transferred to and stored in an MP Biomedicals Lysing E Matrix tube with 0.5 mL of 1× DNA/RNA Shield (Zymo research). A total of three ‘squeezed gut’ samples were collected. Although moderately easy to sample, the samples were inconsistent where a single sample clearly yielded more ‘clean’ faecal matter than did the others.

#### 2.2.3 Intestinal content

The gastrointestinal tract was carefully dissected out and the tip of a single‐use inoculation loop was gently traced along the entire dissected gastrointestinal tract to extrude the intestinal contents avoiding tearing of the intestinal tract, as similarly done in (Gaulke et al., 2016). Using an inoculation loop, ~ ½ of a 10 μL loop of the intestinal content was then transferred to an MP Biomedicals Lysing E Matrix tube with 0.5 mL of 1× DNA/RNA Shield (Zymo research). Four intestinal content samples were collected. Although this method is rather time‐consuming and invasive it has the potential to get both individual‐level variation and potentially more microbial DNA by avoiding host tissue. The four samples were consistent apart from a single sample which yielded substantially more material compared with the others.

#### 2.2.4 Faeces

Faecal matter was sampled directly from the tank using a single‐use polyethylene pipette and transferred to a Petri dish. Using an inoculation loop, ~ ½ of a 10 μL loop, faecal matter was then transferred to and stored in an MP Biomedicals Lysing E Matrix tube with 0.5 mL of 1× DNA/RNA Shield (Zymo research). A total of three replicate faecal samples were collected. This is likely the most prevalent method as it is the easiest and most consistent sample to acquire and completely noninvasive; this sampling method even allows for a time series study to be conducted. However, this approach provides a tank‐level representation of the zebrafish microbiota, lacking individual fish representation. This problem can however be overcome as done by (Gaulke et al., 2019) where individual fish are separated into smaller tanks and faecal matter is subsequently collected.

#### 2.2.5 Water

As environmental control, water samples (*n* = 3) were collected. For each water sample, 500 mL water was collected, 50 mL at a time and filtered using a sterivex filter. Each sterivex filter was then filled with DNA/RNA Shield (Zymo research) before storage. The water samples were stored at −20°C before DNA extraction.

### 2.3 DNA extraction

Prior to DNA extraction, all samples were lysed in a Tissuelyzer (Qiagen) at 30 GHz for 5 min and spun down at 16,000*g* for 5 min. Subsequently, 400 μL lysate from each sample was transferred to a 96 deep well plate in a randomised order. Three DNA/RNA shield (Zymo research) negative extraction controls were included in the process. DNA was extracted following the manufacturer's recommendations of the magnetic bead‐based Quick‐DNA MagBead Plus Kit (Zymo Research). The DNA concentration of each sample was measured using a Qubit fluorometer (Invitrogen).

### 2.4 Shotgun metagenomics library preparation and sequencing

Twenty five microliters of the eluted DNA was shipped to Novogene (Cambridge, UK) for metagenomic shotgun sequencing where DNA was randomly sheared into short fragments by sonication. Library was prepared using Novogene NGS DNA Library Prep Set (Cat No. PT004). The library was quantified with a Qubit fluorometer and real‐time PCR and bioanalyzer 2100 (Agilent) was used for size distribution detection. The quantified libraries were then pooled and subjected to 150 bp paired‐end sequencing on a NovaSeq 6000 instrument (Illumina).

### 2.5 16S amplicon library preparation and sequencing

The remaining 20 μL of DNA was used for generating paired 16S data for all samples. First the V3‐V4 hypervariable region of the 16S rRNA gene was amplified using 341F (5′‐CCTAYGGGRBGCASCAG‐3′) and 806R (5′‐GGACTACNNGGGTATCTAAT‐3) (Yu et al., 2005) with the addition of 20 different tags to the 5′ end of each primer. A negative PCR control was also included. Each reaction was 25 μL and consisted of 13.5 μL ddH_2_0, 2.5 μL 10× TaqGold buffer (GeneAmp®), 2.5 μL Mgcl2 (25 mM), 1.5 μL BSA (20 ng/μL), 0.5 μL dNTPs(10 mM), 0.5 μL DNA polymerase AmpliTaq Gold® (5 U/μL), 2 μL primer mix and 2 μL DNA extract (2 μL ddH20 for the PCR control). The PCR conditions were as follows: Initial denaturation at 95°C for 10 min followed by 30 cycles of denaturation at 95°C for 15 s, annealing at 53°C for 20 s and extension at 72°C for 40 s followed by a final extension at 72°C for 10 min. The amplified PCR products were then pooled in an equimolar fashion based on agarose gel band brightness and purified using the HighPrep PCR Clean‐up System® (MagBio Genomics Inc). Subsequently, the PCR‐free single‐tube metabarcoding library preparation protocol, Tagsteady (Carøe & Bohmann, 2020) was used to construct the library. The library was quantified using the NEBNext® Library Quant Kit for Illumina® (New England Biolabs) by mixing 2 μL 1:10,000 diluted library with 8 μL Quant mastermix with added primers (New England Biolabs). Due to low initial concentration of adapter‐ligated products, we made triplicates of the library, pooled the triplicates, and increased the concentration using a vacuum concentrator, Concentrator plus® (Eppendorf). The library was then sequenced at the GeoGenetics Sequencing Core, University of Copenhagen, Globe institute using an Illumina MiSeq platform, reagent kit v3 at 600 cycles. Negative extraction and PCR controls were included in the library and sequenced.

### 2.6 Bioinformatic processing of shotgun sequencing reads

The quality of raw reads was assessed using FastQC (Andrews, 2010) and MultiQC (Ewels et al., 2016) for subsequent quality‐filtering steps. AdapterRemoval/v.2.3.3 (Schubert et al., 2016) was used for the removal of low quality reads and adapters. Duplicates were removed using the rmdup function in Seqkit/v.2.3.1 (Shen et al., 2016) and reads re‐paired using bbmap/v.38.84 (Bushnell, 2014). Host reads were filtered out by mapping the quality‐filtered reads to the zebrafish genome (GCA_000002035.4) and the potential contamination was removed by mapping to the human genome (hg38) (Schneider et al., 2017) with minimap2/2.24 (Li, 2018, 2021) using default parameters for short accurate genomic reads. All unmapped reads were kept. The mapped reads were kept in separate files to assess the depth of coverage using the samtools‐depth function (Danecek et al., 2021). The metagenomic reads were co‐assembled on a sample type basis (that is, 5 co‐assemblies) using MEGAHIT/v.1.2.9 (Li et al., 2015) with metagenomic sensitive presets and a minimum contig length of 1000 bp. Quality of assemblies was assessed using Quast/v.5.0.2 (Mikheenko et al., 2018). Further analyses of assemblies and visualisation were performed using the anvi'o platform (Eren et al., 2015, 2021).

For each of the five co‐assemblies, the following was done: (i) anvi'o was used to identify Open Reading Frames (ORFs) using Prodigal/v.2.6.3 (Hyatt et al., 2010). (ii) HMMER/v.3.3 (Finn et al., 2011) was used to identify sets of Singe‐Copy‐core Genes (SCGs) of protista, archaeal, and bacterial origin (Lee, 2019). Hidden Markov Models of SCGs were used for estimating the number of recoverable bacterial genomes in the assemblies and for completion and redundancy estimates of MAGs. (iii) The ORFs were annotated in the anvi'o platform using functions from NCBI's Clusters of Orthologous Groups (COGs) (Tatusov et al., 2003), the KOfam HMM database of KEGG orthologs (KOs) (Aramaki et al., 2020; Kanehisa & Goto, 2000) and Pfams (El‐Gebali et al., 2019). (iv) Kaiju (Menzel et al., 2016) was used to infer the taxonomy of genes with NCBI's non‐redundant protein database ´nr´ and import into the anvi'o framework as described here (https://merenlab.org/2016/06/18/importing‐taxonomy/). (v) For the prediction of the number of genomes in the assemblies, we used the ‘anvi‐display‐contigs‐stats’ function. (vi) Reads were then mapped to contigs using minimap2/2.24 (Li, 2018, 2021) and samtools (Danecek et al., 2021) and stored as BAM files. Each BAM file was profiled in anvi'o sung ‘anvi‐profile’ for estimation of coverage and detection statistics of each contig, and each profile was integrated into a merged profile database for further processing using ‘anvi‐merge’. (vii) Finally, Binning was performed using the anvi‐cluster‐contigs which uses CONCOCT/v.1.1.0 (Alneberg et al., 2014). Each bin was then manually refined based on tetranucleotide frequency and differential coverage across samples using ‘anvi‐refine’. MAGs were called using ‘anvi‐rename‐bins’ where each bin that was more than 50% complete and less than 10% redundant was defined as a MAG. Anvi'o was also used to infer MAG taxonomy based on single‐copy core gene sequences from the Genome Taxonomy Database (GTDB) (Parks et al., 2018).

To create a non‐redundant set of MAGs representative of all samples, sequences for each MAG from each merged profile database were extracted. CheckM (Parks et al., 2015) was used as an additional quality assessment of the MAGs. We then applied dRep (Olm et al., 2017) to dereplicate the set of MAGs into a set of non‐redundant species‐level representatives of the collection based on average nucleotide identity (ANI) and quality of MAGs based on the previous CheckM quality assessment (Data S1). We then mapped the metagenomic reads from all samples to the set of non‐redundant MAGs, and followed the same steps as detailed above using the anvi'o platform. For further comparison with existing data, we mapped our metagenomic reads to the Zebrafish Fecal v1.0 MAG catalogue in the MGnify genomes resource (Gurbich et al., 2023).

### 2.7 Bioinformatic processing of 16S amplicon sequencing data

Quality of raw reads from 16S V3–V4 sequencing was assessed using FastQC (Andrews, 2010) and MultiQC (Ewels et al., 2016) for subsequent quality filtering steps. Cutadapt/v.4.4 (Martin, 2011) was used to trim adapter and primer sequences. Subsequently, the DADA2/v1.26 (Callahan et al., 2016) pipeline was applied for quality filtering and trimming, inferring of amplicon sequence variants (ASVs), merging of forward and reverse reads, removal of chimeric sequences, and taxonomy assignment with the SILVA/v138 reference database (Quast et al., 2013). Then Decontam/v1.20.0 (Davis et al., 2018) was applied to remove putative contaminants, after which LULU/v0.1.0 (Frøslev et al., 2017) was applied for ASV curation and removal of erroneous sequences. The ASVs were then merged at the genus level for analyses.

### 2.8 Analyses and visualisation

Rarefaction curves were estimated using the R package *vegan* (Oksanen et al., 2020). The relative abundances of MAGs were calculated using the mean coverage across the MAG and then normalised using the length of the MAG using TPM normalisation with the R package *ADI‐impute* (Xu et al., 2021). Microbiota composition analyses were performed using the R package *phyloseq (*McMurdie & Holmes, 2013). Visualisation of data was performed using the R package *ggplot2* (Wickham, 2016) the anvi'o platform (Eren et al., 2015, 2021). The icons representing sample types, used in figures, were created using BioRender.com.

## 3 RESULTS

### 3.1 Sample types differed in all stages of sample processing

**FIGURE 1:**
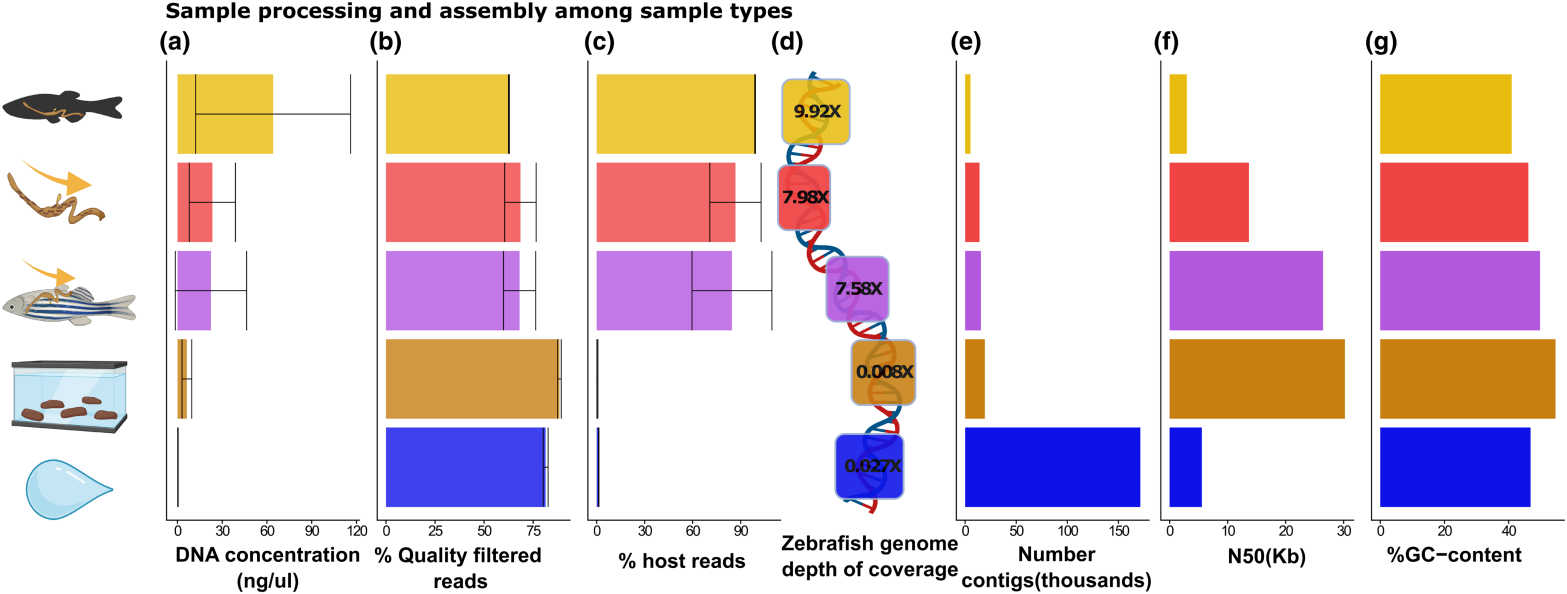
Comparison among sample types through the sample processing. The colours and icons represent different sample types. From top to bottom, they represent whole gut, intestinal content, squeezed gut, faeces, and water. (a) Mean DNA concentration among sample types. (b) Mean percentage of reads removed after quality filtering and deduplication. (c) Mean percentage of quality filtered and deduplicated reads removed after host filtering. (d) Mean depth of coverage of the zebrafish reference genome. (e) Number of contigs in co‐assemblies among sample types (f) N50 value of the co‐assemblies among sample types, and (g) the GC content of the co‐assemblies among sample types.

The sample types differed at every stage of sample processing in data output and quality. Here we refer to sample processing as the process of generating MAGs from DNA extraction through bioinformatic processing and metagenomic assembly. After comparing the practicality of sampling (Table 1), we compared the average post‐extraction DNA concentration among the different sample types (Figure 1a). As expected, due to the high host DNA content, the whole gut had the highest average DNA concentration, followed by intestinal content and squeezed gut, while faeces and water had the lowest average concentration.

Metagenomic shotgun sequencing yielded ~ 2.4 billion reads across 15 samples. A single water sample failed sequencing due to low DNA content along with a negative control (indicating the efficacy of the negative control) and these two samples were therefore not included in the analysis. After quality filtering and removal of duplicates ~ 647.5 million reads remained. The average percentage of reads removed after quality filtering and removal of duplicates was then compared among sample types (Figure 1b). Whole gut samples had the lowest percentage of removed reads followed by intestinal content, squeezed gut, water, and finally most reads were removed from the faecal samples. The standard deviations should also give an indication of the consistency within the sample type group making the intestinal content and squeezed gut less consistent than whole gut and faecal samples. After human and zebrafish filtering ~ 157 million reads remained. The percentage of quality‐filtered reads that were removed by host filtering was then compared among the sample types to give an indication of the amount of host content among sample types (Figure 1c). As expected, the highest amount removed by the host filter was whole gut samples with a strikingly high percentage (99%), followed by intestinal content (86.7%), squeezed gut (84.5%), water (1.29%), and interestingly, the lowest percentage was removed from the faeces samples (0.473%). Notably, the sheer number of reads not mapping to the host zebrafish genome was highest in the water samples followed by faeces, intestinal content, squeezed gut, and finally whole gut. The data therefore counterintuitively indicate that among the sampling types, a lower initial post‐extraction DNA concentration yields more metagenomic reads in the case of zebrafish shotgun metagenomic sequencing.

Despite this, recovering host genetic data may also be a valuable resource for some studies and for avoiding waste of sequencing depth and cost. We therefore included a comparison of the average depth of coverage of the zebrafish genome recovered from each sample type (Figure 1d). Surprisingly, faecal samples presented an unexpectedly lower coverage compared with the water samples. Squeezed gut and intestinal content exhibited relatively similar depths of coverage. As anticipated, the whole gut samples had the highest depth of coverage.

Metagenomic assemblies of the unmapped reads were made for all sample types and compared (Figure 1e–g). The assemblies differed at all levels, the number of contigs was by far the highest in the water samples followed by faeces, squeezed gut, intestinal content, and finally, the lowest number of contigs was observed in the assembly of the whole gut. As depicted (Figure 1f,g), the GC content and N50 value varied widely among the assemblies of the different sample types.

### 3.2 Faecal samples provide the highest number and quality of MAGs among sample types

**FIGURE 2:**
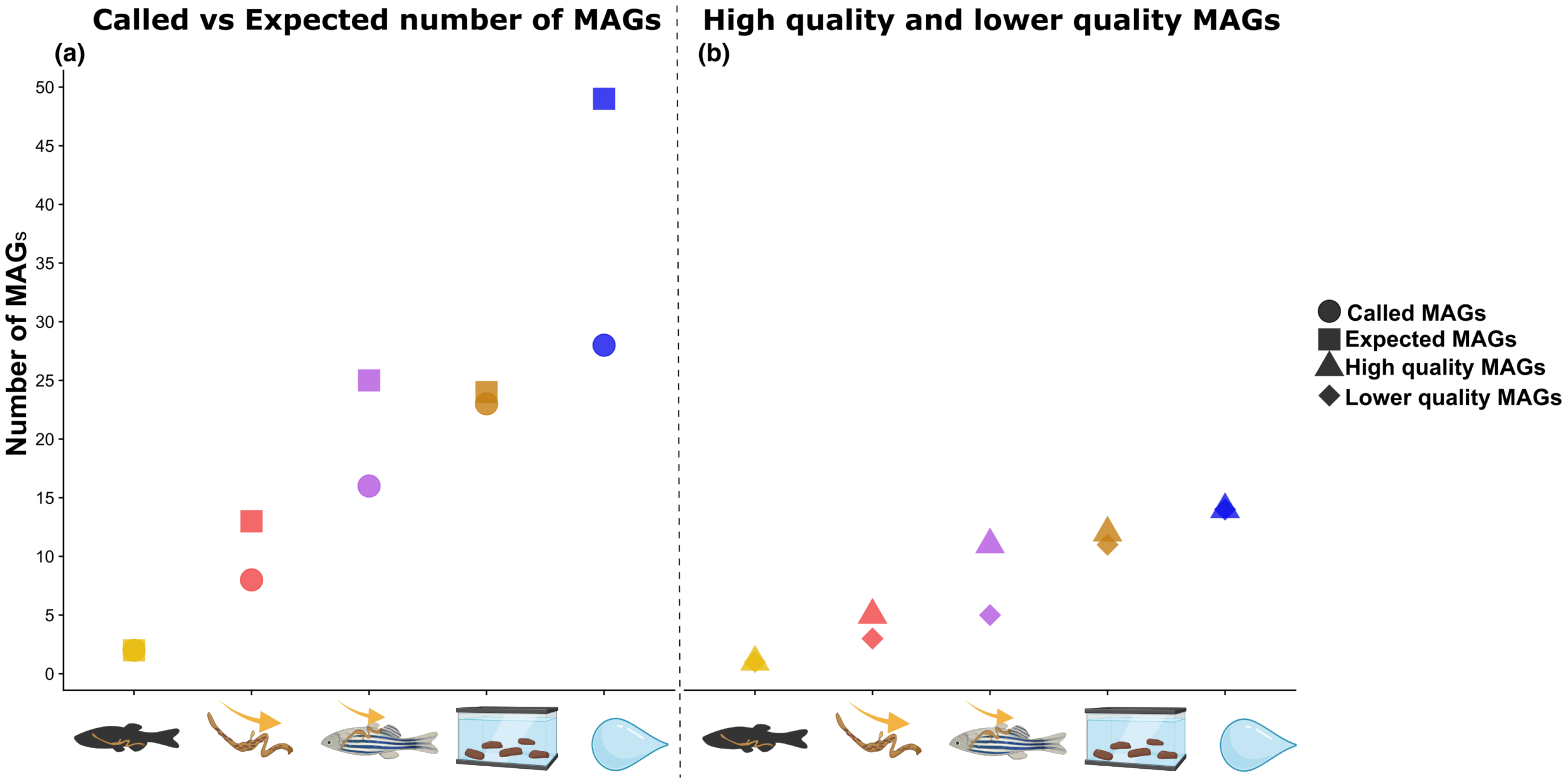
(a) Plot showing the estimated and actual number of bacterial genomes in the co‐assemblies among sample types. The circles represent the number of called MAGs and the squares represent the estimated number of MAGs. (b) Plots showing high‐quality and lower‐quality MAGs. The triangle represents high‐quality MAGs (>90% complete and <%5 redundant) while the diamond represents lower‐quality MAGs (>50% complete and >10% redundant). The colours and icons indicate the sampling type, yellow represents whole gut samples, red represents Intestinal content samples, purple colour represents squeezed gut samples, brown represents faeces and blue represents water samples.

Based on the presence/absence of 71 SCGs, the estimated total number of bacterial genomes in the five assemblies was estimated (Figure 2a). The lowest estimated number of genomes was as expected in the whole gut samples (2 bacterial genomes) followed by, intestinal content (13 genomes), squeezed gut (25 genomes), faeces (24 genomes), and finally water had the highest number of estimated bacterial genomes (49 genomes). After automated binning and manual refinement of the MAGs, we compared the number and quality of MAGs. MAGs were called if they were over 50% complete and had less than 10% redundancy, MAGs were classified as high quality if they were over 90% complete and less than 5% redundant. Low‐quality MAGs less than 50% complete and higher than 10% redundant were not included in the analysis. In total 77, MAGs were called across sample types. The highest number of MAGs (28 MAGs) were called in the water samples (57% of expected MAGs) of which 14 were classified as high quality. A total of 23 MAGs were called from the faecal samples (95% of expected MAGs) of which 12 were classified as high quality. From the squeezed gut samples, 16 MAGs were called (64% of expected MAGs) 11 of which were classified as high quality. Eight MAGs were called in the intestinal content samples (61% of expected MAGs) of which 5 were classified as high quality. Finally, only two MAGs were called in the whole gut samples, one of which was classified as high quality. It is clear that the representativeness, the number and quality of the assemblies vary widely, so to compare the sample types, we created a MAG catalogue consisting of 41 non‐redundant MAGs (see Data S1) and independently mapped host‐filtered reads from all samples back to it.

**FIGURE 3:**
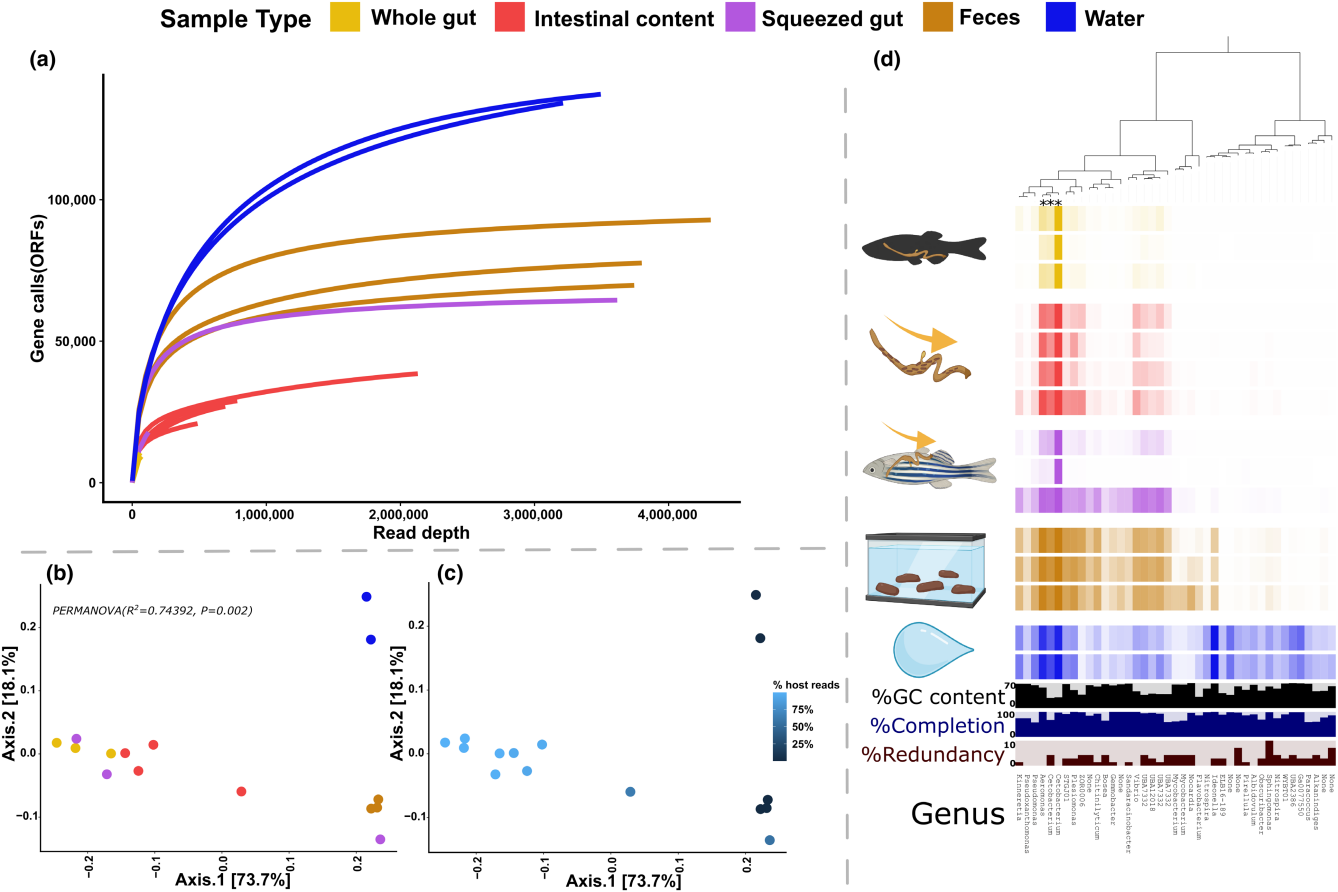
MAG abundance and diversity among sampling types. The colours and icons indicate the sampling type. (a) Rarefaction curve of ORFs among sample types. (b) PCoA of MAG composition among samples using Bray‐Curtis distance with the colours representing sample type as in A. (c) PCoA of MAGs where the blue coloured gradient represents the percentage of removed host reads. (d) log transformed mean per sample coverage of MAGs in the MAG catalogue created for this study. Each column of squares represents a single MAG and each row represents a single sample. GC content of each mag is represented by black bars and completion and redundancy estimates of each MAG is represented by blue and red bars respectively, the genus level of each MAG is also indicated in text. The three MAGs representing over 5% of the overall abundance are indicated with asterixis on the dendrogram.

First, we assessed sequencing depth and generated a rarefaction curve of gene calls across all samples which revealed striking and interesting differences among sample types in terms of data saturation (Figure 3a). Expectedly, as only two MAGs were recovered, the whole gut samples did not reach saturation, indicating insufficient sampling depth. We observed that the intestinal content samples reached saturation indicating sufficient sampling depth. This is in stark contrast to the squeezed gut samples where most of the diversity seems to belong to a single sample while the others do not reach saturation, indicating a lack of consistency in sampling. However, the saturated curve from the single squeezed gut sample is similar to the ones from the faecal samples which all saturate around the same time as do the intestinal content samples although at a much higher diversity. The water samples are almost saturated and indicate the highest diversity. This is supported by the coverage of the MAG catalogue among samples.

We then looked at the relative abundances of taxa across the dataset (Figure 3d). Of the 41 MAGs, only three comprise over 5% of the relative abundance across the sample set, one of which belongs to the genus *Cetobacterium* (assigned species *Cetobacterium sp000799075*) is clearly dominant across all sample types. It comprises the highest percentage in the whole gut samples (85.2%) followed by intestinal content (72.1%), squeezed gut (67.4%), faeces (39.8%), and water (38.7%). The second most abundant MAG belongs to the genus *Aeromonas* and its relative abundance is highest in the faecal samples (13.2%), followed by water (12.4%), intestinal content (11.2%), squeezed gut (5.03%), and whole gut (3.62%). The third and final MAG comprising over 5% of the relative abundance also belongs to the genus *Cetobacterium* (assigned species *Cetobacterium somerae*). Its relative abundance is highest in the faecal samples (8.38%), followed by intestinal content (7.36%), water (6.19%), squeezed gut (6.156%), and finally whole gut (4.04%). A total of 10 MAGs (the three summarised included) comprise over 1% of the cumulative relative abundance (a summary of those can be found in Data S1). It should be noted that one of the 10 MAGs has by far the highest relative abundance in the water samples, that is a MAG belonging to the genus *Ideonella*. Despite the obvious differences among sample types in terms of relative abundance, significant differences were not observed among the sample types, likely due to the small per‐group sample numbers. The beta‐diversity of the microbiota composition shows significant differences based on sample type (Figure 3b). The first axis of the PCoA separates the whole gut, intestinal content, and part of the squeezed gut content from the faecal and water samples and a single squeezed gut sample (indeed the one that looks more like the faecal samples). The second axis of the PCoA separates the water samples reflecting the different composition of the water compared with the other samples. Moreover, there appears to be a gradient in samples ranging from low‐high host DNA content along the first axis of the PCoA (Figure 3c).

**FIGURE 4:**
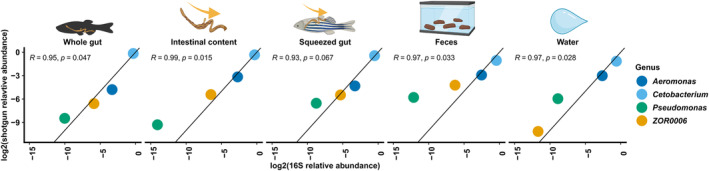
This figure shows the relationship between the log2 transformed relative abundances of the top 4 taxa shared between the 16S metabarcoding data and the shotgun sequencing data among the five sample types. The line represents an *x* = *y* relationship. The icons represent the different sampling types and the colours differentiate the four genera.

Before analysing the functional potential among the sample types we compared the relative abundances of MAGs to 16S amplicon‐based ASVs generated for the same samples (Figure 4) We focused on comparing the relative abundance of four prevalent taxa ensuring a consistent genus‐level match between the 16S dataset and the MAG catalogue. These taxa belong to the genera *Cetobacterium*, *Aeromonas*, *Pseudomonas*, and *ZOR0006*. Although not highly significant in all samples, the correlation between log2 transformed relative abundances of the four taxa in 16S and shotgun seems almost perfect among sample types indicating that the functional metagenomic data accurately represents the composition of the microbiota as characterised by the normally used 16S‐based barcoding approach. The main difference seems to be in the abundances of *Cetobacteriuma* and *Aeromonas* in the faecal and water samples of which there is a slightly higher abundance in the 16S data compared with the shotgun data (see also Supplementary file S1, Figure S1) and in the *Pseudomonas* which is consistently higher in abundance in the shotgun data. Diversity and composition analyses were also performed on the 16S data and they largely reflected the composition of the shotgun data although there was less difference between the sample types, that is, the 16S data from the faecal samples was similar to the whole gut samples (Supplementary file S1, Figure S1).

### 3.3 Functional composition differs among sample types

**FIGURE 5:**
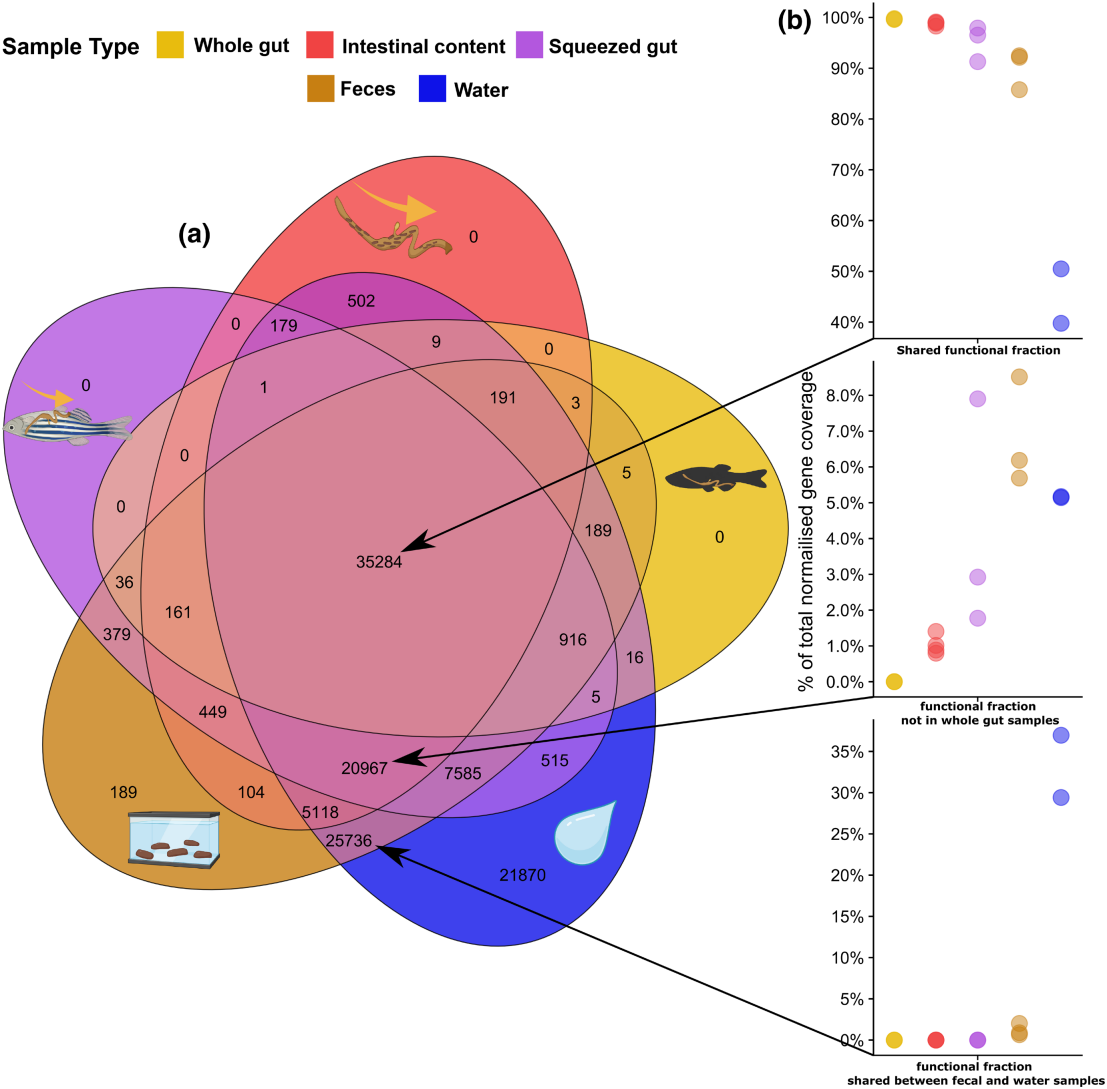
(a) Venn diagram of the number of gene calls with assigned COG categories across sample types. (b) Percentage of normalised gene coverage among sample types in fractions of the Venn diagram. The respective fractions are indicated by arrows. The colours and icons represent the different sample types. The colours and icons indicate the sampling type, yellow is whole gut samples, red represents the intestinal content samples, purple is squeezed gut samples, brown represents faeces and blue represents water samples.

Having established the differences in composition among sample types and validation with 16S metabarcoding data, we aimed to investigate the differences in the functional composition. From the difference between the abundance of MAGs among sample types, we expect the gene content to vary similarly among the sample types with increasing depth of functional inference (Figure 3). We started by comparing COG categories among sample types (Figure 5). A total of 168,366 genes were called across the MAG catalogue and of those 120,410 had assigned COG categories. We compared the sample types in how many gene calls were unique/shared among sample types (Figure 5a) and how much potential functional fraction (that is the fraction of normalised coverage of gene calls) those gene calls contribute (Figure 5b). We identified three main fractions of gene calls among sample types. Firstly, a core fraction of gene calls that are shared among all sample types. Secondly, a fraction shared among all sample types except for whole gut samples was included, likely reflecting the diversity of functions not captured by the whole gut content samples due to high host DNA content. And lastly, a fraction is shared exclusively between faecal samples and water samples. All sample types shared a core fraction of 35,282 gene calls with assigned COG functions. This fraction of gene calls accounts for most of the potential functional abundance 99.7% (SD = 0.0974) in the whole gut samples, 98.8% (SD = 0.359) in the intestinal content samples, 95.2% (SD = 3.49) in the squeezed gut samples, 90.1% (SD = 3.77) in the faecal samples and only 45.1% (SD = 7.58) in the water samples suggesting that these functions likely represent a set of core functions in the zebrafish gut microbiota opposed to be sourced from the water microbial community. In the fraction shared among all samples except the whole gut samples (20,967 gene calls), the fraction of functional potential was only 1.02% (SD = 0.269) in the intestinal content samples, 4.2% (SD = 3.26) in the squeezed gut samples, 6.79% (SD = 1.51) in the faecal samples and 5.16% (SD = 0.0203) in the water samples. Thus, likely reflecting the increased resolution of the microbial functions of lower abundance taxa when avoiding sampling of the host gut tissue. Looking at the fraction, only shared among the water and the faeces (25,736 gene calls), it accounts for only 1.19% (SD = 0.728) of the functional potential in the faecal samples in stark contrast with the 33.2% (SD = 5.34) in the water samples. This likely reflects a fraction which corresponds to contamination from the water samples. We then removed the fraction of gene calls from the sample set that had a higher mean coverage in the water samples compared with the other sample types. This resulted in a big reduction of shared gene calls both in the fractions shared among only the faecal samples and water samples and in the fraction shared among all sample types apart from whole gut samples. Further addressing this, we focused only on the gene calls with COG functions from the 10 MAGs that constituted over 1% of the relative abundance (24,432 gene calls). Subtracting the gene calls from the *Ideonella* MAG, which had the highest abundance in the water samples completely removed the fraction of gene calls (2604 gene calls) shared between only the faecal and water samples along with a part of the fraction shared between all sample types except the whole gut samples. This analysis was repeated for gene calls with assigned Pfam functions (Supplementary material file S1, Figure S2) and indicated similar results.

### 3.4 Intra‐MAG variability is driven by MAG coverage

**FIGURE 6:**
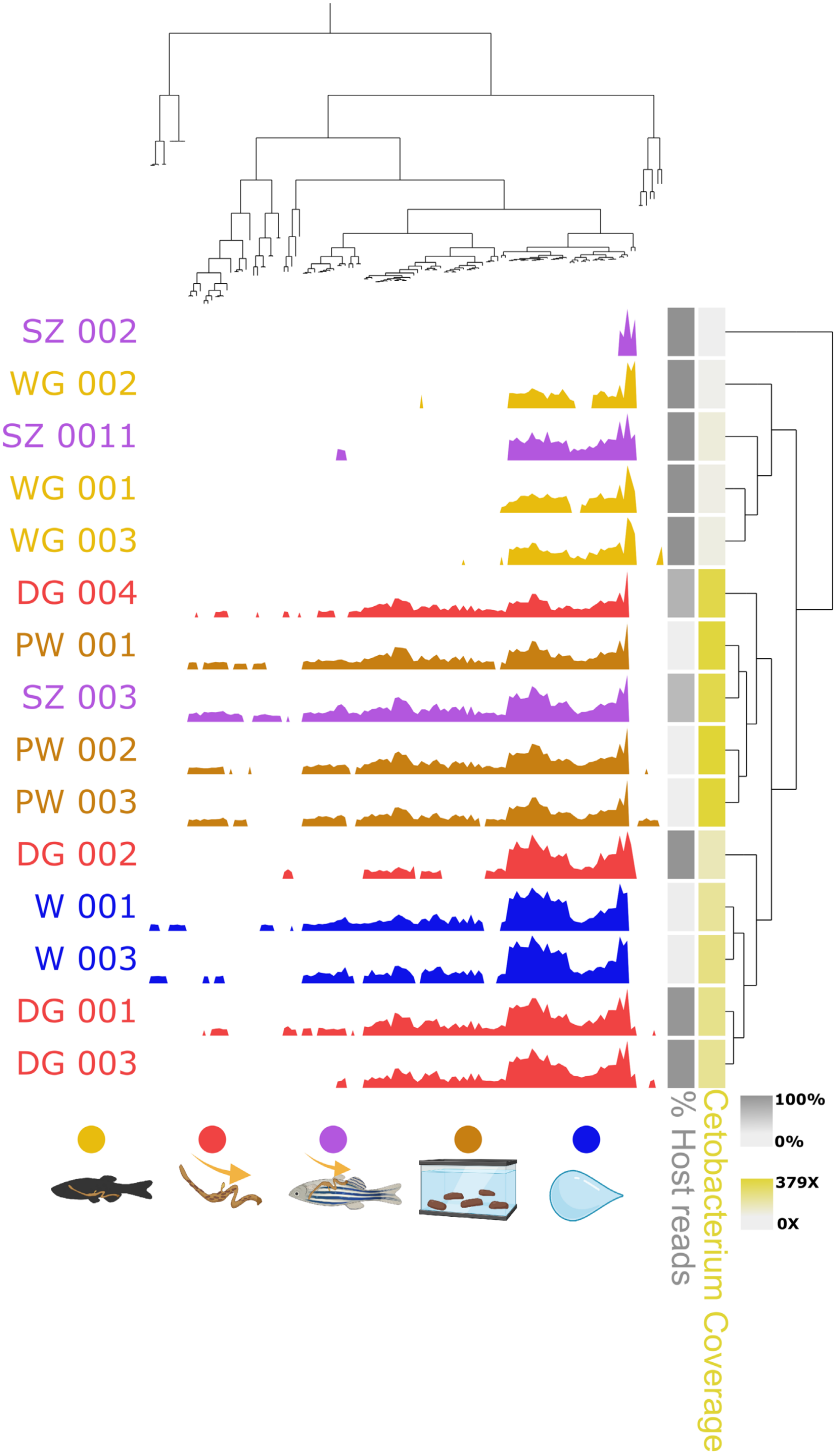
This figure shows the variability of single‐nucleotide variants (SNVs) in the high abundance Cetobacterium sp000799075 MAG. SNVs with a minimum coverage of 10× across 90% of the samples were taken into account. The variability of SNVs is hierarchically clustered using ward ordination and Euclidean distance based on the similarity of variability. The vertical ordering is clustered based on similarity among samples. The two columns on the right represent a gradient of the MAG's coverage in each sample (yellow) and a gradient of percentage of host reads in each sample.

To briefly address the increasingly insightful and rising topic of microbial population genetics, we profiled the single‐nucleotide variants (SNVs) variability among sample types (Figure 6). To do this, we chose the highest abundant MAG across the sample set, namely, the *Cetobacterium sp000799075* MAG. Only SNVs with more than 10X coverage across 90% of the samples were included. The results indicate that the degree of variability is mainly driven by the coverage of the *Cetobacterium sp000799075* MAG (Pearson correlation *R* = .91, *p* = 1.9 × 10^−6^). That is, the higher the MAG coverage (and the lower the host DNA content depending on sample type), the more variation is recovered for each SNVs indicating that too low coverage of MAGs leads to loss of information about the intraspecific microbial population diversity.

### 3.5 The representation of MAG catalogues differ among sample types

**FIGURE 7:**
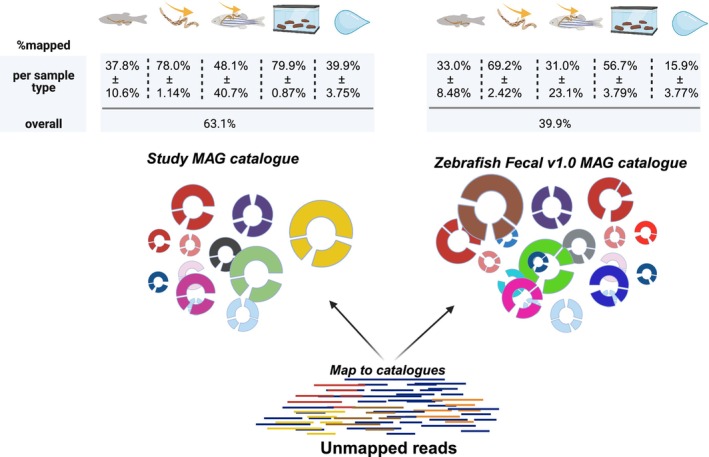
This figure shows the comparison of unmapped reads generated in this study mapped to the non‐redundant MAG catalogue generated for this study and the Zebrafish Fecal v1.0 MAG catalogue from (Gurbich et al., 2023) which was generated by publicly available zebrafish shotgun metagenomic sequences. Figure created using BioRender.com.

We also wanted to investigate how the data generated for this study compared with previously generated zebrafish metagenomic data. We mapped our metagenomic reads to a previously curated MAG catalogue (Gurbich et al., 2023; Richardson et al., 2023) consisting of 101 MAGs. We compared the fraction of metagenomic reads mapping to the Zebrafish Fecal v1.0 MAG catalogue to the percentage mapping to our own MAG catalogue which should estimate both the representativeness of both catalogues with regards to our data and sample types (Figure 7). The overall trend among all sample types was that a higher percentage of reads mapped to the MAG catalogue generated for this study compared with the reference Zebrafish Fecal v1.0 MAG catalogue, with the difference ranging between 5% and 24%. Notably, the highest fraction of reads mapped to both catalogues was observed in the intestinal content samples followed by faecal samples, squeezed gut samples, whole gut samples, and finally the water samples.

## 4 DISCUSSION

We aimed to illustrate the impact of sampling technique on bioinformatic processing and analyses of teleost gut microbiota through a typical genome‐resolved metagenomic pipeline. We considered the zebrafish model and compared four sampling techniques as well as water samples serving as an environmental control to assess the potential environmental contamination from water‐related microbes. We compared the sample types on practicality and consistency of sampling, through quality filtering, assembly, MAG recovery, and microbiota composition.

Considering a sample that constitutes a good representative for the zebrafish gut microbiota, sampling the whole gut seemed like a good idea at a first glance since in theory, it should capture the entirety of what is in the gut and even potential intracellular gut bacteria without potential contamination from the environment. However, considering the results, it may not be the best representative sample. The sheer amount of host reads (99%) leads to low number of microbial reads which leads to problems with downstream analyses. Even though we sequenced 20G per sample, the sequencing depth of the microbial content of the whole gut samples was insufficient. We only recovered two MAGs, which is low compared with the rest of the sample types and what we expected from existing literature (Kayani et al., 2021, 2022). Those MAGs, belonging to the genera of *Cetobacterium* and *Aeromonas*, were however the ones that dominated the relative abundance across all sample types. This may indicate that despite a low metagenomic content in the whole gut sample, the potential for monitoring the dominating bacteria in the gut is there. We encountered similar problems with sampling the intestinal content, although to a lesser extent.

The content of the zebrafish intestinal tract should, like the whole gut samples, provide a complete representation of the zebrafish gut microbiota. Although more time‐consuming in time spent per sample, the intestinal content had a lower amount of host DNA than the whole gut samples. Although 87% host DNA is a large fraction, it is an improvement from the whole gut samples. Rarefaction analyses indicated sufficient sequencing depth and saturated curves although the diversity is less than the faecal samples. Despite the overall higher quality of data from the intestinal content samples compared with the whole gut samples, the microbiota profiles largely reflect a similar composition to the whole gut samples, if the non‐redundant MAG catalogue is followed, with Cetobacterium and Aeromonas dominating the composition. Overall, the intestinal content samples seem to provide a microbiota resolution that is an intermediate between whole gut and faecal samples.

We included squeezed gut samples as we thought they would be a potentially good alternative to faecal samples. That is, sampling the faeces from the fish without dissecting out the gut and without the faeces coming into contact with the surrounding water, thus largely eliminating the potential problem for host and environmental contamination. However, according to the results, unfortunately, the sampling was inconsistent. Analyses of two of the three samples taken indicated insufficient sequencing depth and a similar host content as in the whole gut samples, in fact, they largely reflected the whole gut samples throughout the bioinformatic processing and analyses. However, a single sample only had 55% host content, indicating sufficient sequencing depth and larger diversity compared with the intestinal content samples. This sample is likely responsible for the majority of the 16 MAGs recovered from the binning efforts of assembled contigs from the squeezed gut samples. We believe that if this sampling method can be improved in terms of consistency, it lessens the risk associated with potential environmental contamination as in the faeces sampled directly from the tank water. Interestingly, the squeezed gut sample with higher diversity largely reflected the composition and diversity of the faecal samples. That sample may therefore represent the microbial fraction not present in the whole gut and intestinal content samples while avoiding the potential water contamination.

Sampling faeces directly from the tank is the most prevalent method that has been used in the existing literature of shotgun metagenomic sequencing of the zebrafish microbiota (Gaulke et al., 2020; Kayani et al., 2021, 2022). The sampling effort indicated very low host contamination (<1%), sufficient sequencing depth and recovery of 23 MAGs, moreover, the three replicate samples were largely consistent throughout the analyses. The faecal samples seem to reveal a microbial fraction not present in the whole gut or intestinal content samples. In this study, the faecal samples were not from individual fish but rather represent a group level within each tank. Although potentially impacting the comparisons among the sample types, the results should be applicable to individual‐based sampling efforts. For obtaining an individual resolution (Gaulke et al., 2019) provide a solution where individual fish were placed in individual tanks overnight and faecal matter sampled. Whether such a method produces an additional discernible tank effect should be investigated. In terms of the faecal sampling strategy used in this study, there are more methods that can be easily applied depending on resources, time, and purpose, such as collecting the faeces from the water, spinning down the sample and removing the supernatant leaving a pellet that can be processed as a faecal sample as done in (Sieler Jr et al., 2023), Although the initial faecal sampling methodology may thus slightly impact the results in terms of quality, considering the low amount of host contamination and that the end result is a faecal sample without host tissue, the results presented here are suitable for other faecal sampling strategies The faecal samples analysed here raised two questions, one regarding the composition of the microbiota, as it was discernibly different from the other samples and secondly regarding environmental contamination.

Starting with the overall microbiota composition it is clear that two MAGs dominate the microbiota composition (the *Cetobacterium sp000799075* MAG and *Aeromonas* MAG), These have been found in high abundance in both previous shotgun metagenomic studies and in 16S‐based studies. This study may simply reflect the microbiota of the zebrafish in the facilities in which the study was conducted, as facility/study‐specific microbiota are well documented (Roeselers et al., 2011; Sharpton et al., 2021). What is more interesting is the difference in composition and diversity between the intestinal content samples and the faecal samples. This might simply reflect the added microbial fraction not captured by the intestinal content samples. It could also be that the faecal samples include microbes that are simply passing through. Otherwise, it is a possibility that the faecal samples and the intestinal content samples just represent different ‘niches’ or different parts of the zebrafish gut microbiota. The number of MAGs generated in this study is perhaps at first glance rather low compared with the existing zebrafish literature, but considering the high host content and the abundance of the most abundant MAGs, this is perhaps to be expected. However, future studies are expected to have larger sample sizes compared with this pilot, and should improve the binning efforts and yield a higher number of MAGs. On the other hand, a similar low number of MAGs have been reported in other metagenomic studies of fish species like Atlantic cod (Le Doujet et al., 2019) and Atlantic Salmon (Cheaib et al., 2021; Rasmussen et al., 2023), seemingly reflecting a general trend of low functional diversity in teleost gut microbiomes (Limborg et al., 2023).

One of the main concerns and basis of this study was the potential contamination of faecal samples from the surrounding water. We therefore attempted to apply a functional approach to the estimation of the extent of contamination. In terms of functional abundance, the results indicated a low amount of contamination in the faecal samples. The results also indicate that the water samples are more contaminated from the faecal samples than the faecal samples are potentially contaminated by the water samples. This may be the case due to a higher biomass in the faecal samples, potentially overwhelming the water microbiota community. One might also assume that MAGs or genes with higher coverage in the water samples should originate from the water; however, one can never exclude the possibility that a higher coverage MAG from the water lives in a lower abundance niche in the zebrafish gut. Therefore, it is difficult to say whether actual water contamination occurs in the faecal samples, or if it is simply something that is present in low abundance in the faecal samples. Furthermore, it is difficult to say whether increased sample numbers and sequencing efforts would potentiate or reduce this contamination risk. These results are based on coverage of genes and thus any analytical approach using presence/absence of genes or metabolic pathways need to be further scrutinised as a large fraction of gene calls is shared between the environmental water microbiota (Figure 5a) and the intestinal sample types. Surprisingly, more host DNA contamination was present in the water samples than the faecal samples, making the use of water samples as a no‐host control potentially inappropriate. The host content in the water from the tank may potentially originate from shedded mucus or other tissues from the fish. Despite the low potential water contamination of the faecal samples, taken together the results indicate that caution should be taken when interpreting results from faecal matter sampled directly from the tank, and that water samples serve as an important environmental control that can help identify and remove MAGs more likely to be sourced from the water.

We further compared our non‐redundant MAG catalogue with a previously published MAG Zebrafish faecal catalogue (Gurbich et al., 2023) consisting of 101 MAGs. Overall, the MAG catalogue generated in this study performed better compared with the existing reference catalogue. This is not surprising as our study catalogue was generated from our data and should thus suit it better. However, the difference between the percentages of reads mapping back to the catalogues was smaller than expected. This demonstrates the usefulness of using other reference MAG catalogues, as having a comprehensive MAG catalogue can make data processing and analyses more straightforward. One may for example capture a higher fraction of reads for downstream analyses otherwise lost in the local assembly process where rare species may not be well represented in study‐specific de novo MAG catalogues. This is particularly relevant to zebrafish considering the facility and study‐related differences documented in the 16S zebrafish microbiota literature (Roeselers et al., 2011; Sharpton et al., 2021).

The number of MAGs recovered per sample type is relatively low compared with existing literature in zebrafish (Gaulke et al., 2020; Gurbich et al., 2023; Kayani et al., 2021, 2022). However, considering the limited per‐group sample number it is expected that the number of MAGs will increase with increased sample size due to increased binning power. The small per group sample sizes of two to four and only 15 samples in total, led us to largely compare the sample types qualitatively. We decided on a small‐sample‐size‐deeper‐sequencing instead of larger‐sample‐size‐shallower‐sequencing approach since we expected a high host DNA content in most of the sample types, based on previously published literature of fish intestinal microbiota studies (Collins et al., 2021; Hennersdorf et al., 2016; Rasmussen et al., 2021; Riiser et al., 2019).

The amount of host DNA is clearly different among the sample types, resulting in lower resolution of the microbial diversity. However, this is not necessarily detrimental. If there is combined interest in the host genome and microbiota, sequencing the whole gut or the squeezed gut may be a good option to retrieve whole host genomes along with the microbial fraction in one sequencing run (Marcos et al., 2022).

In this study, all samples were collected from adult zebrafish. Thus, the study does not cover sampling methodology for shotgun metagenomic sequencing in other life‐stages such as larval zebrafish. Obtaining metagenomic data for the larval fish is a different challenge entirely; therefore, it is unlikely that any of the sampling strategies discussed here would be sufficiently applicable to larval zebrafish, except for whole intestinal samples which can be dissected from the larvae (Rawls et al., 2004; Stagaman et al., 2020). However, this would then likely, similarly to the results presented here, result in a high host DNA content, and low recovery of microbial reads. While larval zebrafish, with their unique sampling challenges, offer invaluable insights into early development of the microbiota, we could see an expansion of focus on the adult zebrafish microbiota as a stable and complementary counterpart in understanding the microbial community dynamics. Although other life‐stages were not addressed in this study, the results presented are likely applicable to juvenile and adult zebrafish along with other small fish species or, for example, amphibians with similar exothermic lifestyles.

For studies simply aiming to study the zebrafish microbiota composition and function, the amount of host DNA in the host‐derived samples is an obvious problem. A simple approach to increase the resolution of the microbiota is to increase the sequencing depth. However, this is poorly scalable, in terms of cost, sample number, data produced, and computational resources. Other methods to reduce host contamination such as chemical host depletion pre or post DNA extraction, microbial enrichment, and host depletion by adaptive sequencing have been applied and compared with varying degrees (Horz et al., 2008; Marotz et al., 2018; Marquet et al., 2022; Yap et al., 2020). These promising alternatives to deep‐sequencing approaches are interesting tools to apply to intestinal zebrafish samples for shotgun or long‐read metagenomic sequencing. As this study simply aimed to compare and evaluate sample types these approaches were not tested. Overall, this study indicates that the choice of sample type matters and underscores the importance of environmental controls, which allows for more scrutiny in analyses. We have attempted to summarise the results from the study (Table 2) with the aim to guide researchers planning to undertake genome‐resolved shotgun metagenomic approaches in zebrafish or other teleost species. We conclude that the method of choice should depend largely upon the aim of the study and that special care has to be taken concerning environmental contamination.

**TABLE 2:** The table summarises the main results. The colours of the cells correspond to degrees of comparison. Green represents the most favorable ranking, yellow represents the moderate ranking and red represents the least favorable ranking. The light‐red represent the least favourable ranking in the conditions of this study but indicates that there is potential for alternative solutions or optimisation.

	Dissected whole gut 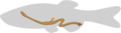	Dissected intestinal content 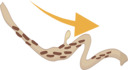	Squeezed gut content 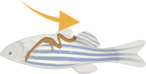	Faeces 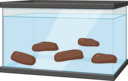
Sample description	Whole gut dissected from fish	Whole gut dissected from fish and intestinal content dissected out	Pressure applied to the abdominal side of the fish from the pectoral fin to the anal fin and the excretion sampled	Faecal matter collected from the bottom of the tank
Invasiveness	High	High	Moderate	None
Ease of sampling	Moderate	Difficult	Moderate	Easy
Consistency	High	High	Low^a^	High
Sample time	Long	Long	Moderate	Short
Individual resolution	Yes	Yes	Yes	No^b^
Host dna content	High	High	High^a^	Low
Saturated rarefaction curve	No	Yes	Partially^a^	Yes
Mag recovery	Poor	Moderate	Moderate	Good
Water contamination susceptibility	Low	Low	Low	Moderate

^a^ Potential for optimisation and improvement in sampling strategy.

^b^ Can be used for individual resolution as described by (Gaulke et al., 2019).

## AUTHOR CONTRIBUTIONS

**Eiríkur A. Thormar:** Conceptualization (equal); data curation (equal); formal analysis (equal); investigation (equal); methodology (equal); software (equal); visualization (equal); writing – original draft (lead); writing – review and editing (equal). **Søren B. Hansen:** Conceptualization (equal); data curation (equal); formal analysis (equal); software (equal); writing – review and editing (equal). **Louise von Gersdorff Jørgensen:** Conceptualization (supporting); data curation (equal); project administration (equal); resources (equal); supervision (equal); writing – review and editing (equal). **Morten T. Limborg:** Conceptualization (equal); data curation (equal); funding acquisition (equal); investigation (equal); resources (equal); supervision (equal); writing – original draft (equal); writing – review and editing (equal).

## CONFLICT OF INTEREST STATEMENT

The authors have no competing interests to declare.

## BENEFIT SHARING STATEMENT

Benefits from this research accrue from the sharing of our data and results on public databases as described above.

## Supporting information


Figure S1‐S2.

Data S1.

## Data Availability

All raw sequences generated for this study in the European Nucleotide Archive (ENA) under the accession number PRJEB71469 (https://www.ebi.ac.uk/ena/browser/view/PRJEB71469) (Thormar, 2023a). An overview of all MAGs generated for the study is publicly available at (https://doi.org/10.6084/m9.figshare.24866772.v1) (Thormar, 2023b). along with fasta files for all MAGs at (https://doi.org/10.6084/m9.figshare.24866790.v1) (Thormar, 2023c), including the non‐redundant set of MAGs at (https://doi.org/10.6084/m9.figshare.24866841.v1) (Thormar et al., 2023). Code used for analysing and processing the data can be found at https://github.com/eirikurandri/Microzebra.
